# Neuronal cell differentiation of mesenchymal stem cells originating from canine amniotic fluid

**DOI:** 10.1007/s13577-013-0080-9

**Published:** 2013-10-29

**Authors:** Eun Young Kim, Kyung-Bon Lee, Jung Yu, Ji Hye Lee, Keun Jung Kim, Kil-Woo Han, Kang-Sun Park, Dong-Soo Lee, Min Kyu Kim

**Affiliations:** 1 Laboratory of Animal Reproduction and Physiology, Department of Animal Science and Biotechnology, College of Agriculture Life Science, Chungnam National University, Daejeon, 305-764 Republic of Korea; 2 Department of Regeneration and Advanced Medical Sciences, Graduate School of Medicine, Gifu University, Gifu, 501-1193 Japan

**Keywords:** Amniotic fluids, Mesenchymal stem cells, Degenerative diseases, Canine, Neural precursor, Neurodegenerative diseases

## Abstract

The amniotic fluid contains mesenchymal stem cells (MSCs) and can be readily available for tissue engineering. Regenerative treatments such as tissue engineering, cell therapy, and transplantation show potential in clinical trials of degenerative diseases. Disease presentation and clinical responses in the* Canis familiaris* not only are physiologically similar to human compared with other traditional mammalian models but is also a suitable model for human diseases. The aim of this study was to investigate whether canine amniotic-fluid-derived mesenchymal stem cells (cAF-MSCs) can differentiate into neural precursor cells in vitro when exposed to neural induction reagent. During neural differentiation, cAF-MSCs progressively acquire neuron-like morphology. Messenger RNA (mRNA) expression levels of neural-specific genes, such as* NEFL*,* NSE*, and* TUBB3* (βIII-tubulin) dramatically increased in the differentiated cAF-MSCs after induction. In addition, protein expression levels of nestin, βIII-tubulin, and tyrosine hydroxylase remarkably increased in differentiated cAF-MSCs. This study demonstrates that cAF-MSCs have great potential for neural precursor differentiation in vitro. Therefore, amniotic fluid may be a suitable alternative source of stem cells and can be applied to cell therapy in neurodegenerative diseases.

## Introduction

Prior to new medical treatments being approved for regenerative medicine, clinical trials in animal models are essential [1–3]. A number of researchers demonstrate that* Canis familiaris* is a suitable model for human diseases [4, 5]. First, humans and dogs share the environmental life pattern. Physiologically, disease presentation and clinical symptom of the dog are much more similar to those of the human compared with other traditional animal models, and half of the canine diseases also belong to the group of human diseases [5]. In the gene database of inherited disorders and traits in >135 animal species, the dog has the most important potential as an animal model for human disease. Also, genomically, the genetic bases of disease susceptibility, morphological variation, and behavioral traits in dogs are significantly related with humans [6, 7]. Therefore, * C. familiaris* could be a promising animal model for human regenerative medicine.

Mesenchymal stem cells (MSCs), generally known as adult stem cells, were first isolated from postnatal bone marrow [8], and its presence was identified in most mammalian tissues. Principal sources of MSCs are bone marrow, umbilical-cord blood, olfactory bulb, amniotic fluid (AF), and Wharton’s jelly [9, 10]. As MSCs derived from AF (AF-MSCs) have the potential for self-renewal and multipotency, they are also defined as stem cells. AF-MSCs are easily isolated and demonstrate multiple differentiation abilities [11–15]. AF-MSCs can be differentiated into chondrogenic, adipogenic, osteogenic, myogenic, endothelial, and neurogenic pathways, as with other MSC sources [14, 16–22]. However, the difference between embryonic stem cell and other MSC sources is that AF stem cells have anti-inflammatory, low immunogenic characteristics; display nontumorigenicity; and there are few ethical issues surrounding their clinical application [23–25]. For those reasons, AF cells have advantages as materials for regenerative medicine.

Stem-cell-mediated therapy is a potential clinical treatment for degenerative diseases. One type of degenerative disease, neurological disorders, has limited treatment because the condition of the nervous system cannot be completely recovered after damage [26, 27]. To improve treatment of neurodegenerative disorders, MSC-mediated cell therapy and transplantation has been studied during the past two decades [28]. Many researchers demonstrate that stem cells can be differentiated into neural precursor cells [18–20, 29, 30], and their use in clinical treatments has been tried.

The aim of tis study was to investigate whether MSCs originating from canine AF (cAF-MSCs) can differentiate into neural precursor cells by using an identical neural induction reagent. We also examined whether differentiated neural cAF-MSCs have the characteristics of dopaminergic cells that secret the dopamine neurotransmitter to prevent Parkinson’s disease.

## Materials and methods

Unless otherwise stated, all chemicals used in this study were purchased from Sigma-Aldrich Chemical Co. (USA). The protocol for this research was approved by the Research Ethics Committee of Chungnam National University.

### Isolation and culture of canine amniotic-fluid-derived cells

Canine AF cells were characterized to MSCs by the method described previously [31]. Briefly, canine AF was collected from cesarean section by centesis under ultrasonographic guidance. The collected AF was centrifuged at 3,000 rpm for 10 min, and the pellet was washed twice with phosphate-buffered saline (PBS, Gibco). Isolated AF cells were seeded into a 60-mm culture dish containing low-glucose Dulbecco’s modified Eagle medium (L-DMEM) supplemented with 10 % fetal bovine serum (FBS, Gibco), 5 ng/ml fibroblast growth factor (FGF), 10 ng/ml epidermal growth factor (EGF), and 0.1 % penicillin–streptomycin (500 U/ml penicillin–5 mg/ml streptomycin, P4458) at 39 °C, 5 % carbon dioxide (CO_2_) in air for 4–5 days.

### Neural precursor differentiation of cAF-MSCs

For preneural differentiation, cAF-MSCs were cultured in L-DMEM supplemented with 10 % FBS, 1× N2-supplement (Gibco), 10 ng/ml FGF, 10 ng/ml EGF, and 0.1 % penicillin–streptomycin. Two days after cultivation of preneural differentiation, the preinduction media was removed and cultured in L-DMEM supplemented with 0.5 % FBS, 1x N2 supplement, 1 mM N6.2′-0-dibutyryl cyclic adenosine monophosphate (db-cAMP), 200 μM butylated hydroxyanisole (BHA), and 0.1 % penicillin–streptomycin at 39 °C, 5 % CO_2_ in air for 5 days.

### Morphological analysis

To clearly observe cell phenotype, cAF-MSCs were washed in PBS and stained using Diff-Quik kit (Sysmax Corporation, Japan), as recommended by the manufacturer’s instructions. Briefly, cells were fixed by Diff-Quik fixative solution and stained by Diff-Quik solution I and II. The stained cells were observed using fluorescence microscope (TE2000-U, Nikon, Japan).

### RT-PCR analysis

Total RNA (tRNA) was extracted using RNA extract kit (MACHEREY–NAGEL, Germany), as recommended by the manufacturer’s instructions. RNA samples (1 μg tRNA) were primed with oligo dT primer to synthesize complementary DNA (cDNA) using iScript reverse transcriptase (Bio-Rad, USA). Real-time polymerase chain reaction (RT-PCR) reaction cycle consisted of a 3-min denaturation at 94 °C, followed by 34 cycles at 94 °C for 30 s, 60 °C for 30 s, and 72 °C for 30 s. A final extension was completed at 72 °C for 5 min, and RT-PCR reactions were modified to 35 cycles with primer annealing at 58–60 °C. RT-PCR-specific amplification of target genes was confirmed by DNA sequencing. Results were evaluated by Image J (National Institute of Health, USA) and normalized to βII-microglobulin (β-MG). Primers are listed in Table 1.

**Table 1:** Real-time polymerase chain reaction (RT-PCR) primer sequences for canine-neural-specific genes

Gene	Ref sequence	Primer sequence (5′-3′)	Annealing temperature (°C)	Size (bp)
*NEFL*	NC_006607	Forward GAAGAAGCTGCCAAGGAAGA	58	202
		Reverse GTTGACCTGATTTCGGGAGA		
*GFAP*	NC_006591	Forward ACCTGGCCAGTTATCGACAG	58	206
		Reverse TCTTAGGGCTGCTGTGAGGT		
*NSE*	NC_006609	Forward GAGAACAGTGAAGCCTTGGA	60	298
		Reverse ACCAATCTGGTTGACCTTGA		
*TUBB3*	NC_006587	Forward GCACACTGCTCATCAACAAG	60	227
		Reverse TCTTGCTCTCCTTCATGGAC		
*B2M*	NC_006612	Forward TCTACATTGGGCACTGTGTCAC	58	136
		Reverse TGAAGAGTTCAGGTCTGACCAAG		

### Immunocytochemistry analysis

Cells were rinsed three times in PBS and fixed with 4 % paraformaldehyde (4 g paraformaldehyde in 100 ml PBS) for 15 min, followed by permeabilization with 0.1 % Triton-X 100 in PBS for 20 min. Cells were then blocked and incubated in rabbit anti-nestin antibody (Abcam, MA, USA) diluted 1:200, rabbit anti-βIII-tubulin antibody (Abcam) diluted 1:200, and rabbit anti-tyrosine hydroxylase (TH) antibody (Abcam) diluted 1:1000 in PBS containing 3 % bovine serum albumin (BSA) overnight. Cells were washed three times again in PBS containing 1 % Tween-20 and incubated with secondary immunoglobulin (Ig)G rabbit antibody (Abcam) diluted 1:200 in blocking solution for 40 min before final washing. The neuronal protein markers were detected under a confocal laser-scanning microscope (LSM5 live configuration Vario Two VRGB), processed with electronics and computer module (Real Time control system), and standard software (system configuration, ReUse function, acquisition, Z-function multitracking, presentation, image operation, fast-focusing system, etc.). At least ten fields of view from three to five independent experiments were used to count nestin, βIII-tubulin, and TH-positive cells: 4′0.6-Diamidino-2-phenylindole (DAPI, Life Technologies, CA, USA), which has a high affinity specifically for DNA, was used for nuclear staining to count total cell number.

### Fluorescent-activated cell-sorter (FACS) analysis

Cells were harvested from the culture dish, centrifuged, resuspended in PBS, and fixed with 4 % paraformaldehyde and permeabilized with 0.1 % Triton-X 100. Neuronal cell markers were detected by anti-rabbit nestin antibody, β-tubulin III antibody, and TH antibody, followed by fluorescein-isothiocyanate-conjugated secondary antibody (Abcam) diluted 1:100. Concentrations of nestin, β-tubulin III, and TH antibody were described, as above. Results were analyzed using the fluorescent-activated cell-sorter (FACScan) instrument operating with CELLQuest software (Bencton Dickinson, USA).

### Statistical analysis

Statistical data are presented as mean ± standard error of the mean (SEM). Significant differences among samples were determined using the JMP 10 statistics program (SAS Institute, Inc., Cary, NC, USA). Comparisons for each pair or all pairs were analyzed using Student’s *t* test or Tukey–Kramer honestly significant difference test. Values of *P* < 0.05 were considered to be significantly different.

## Result

### Morphological changes on differentiation of cAF-MSCs into neural precursor cell

**Fig. 1:**
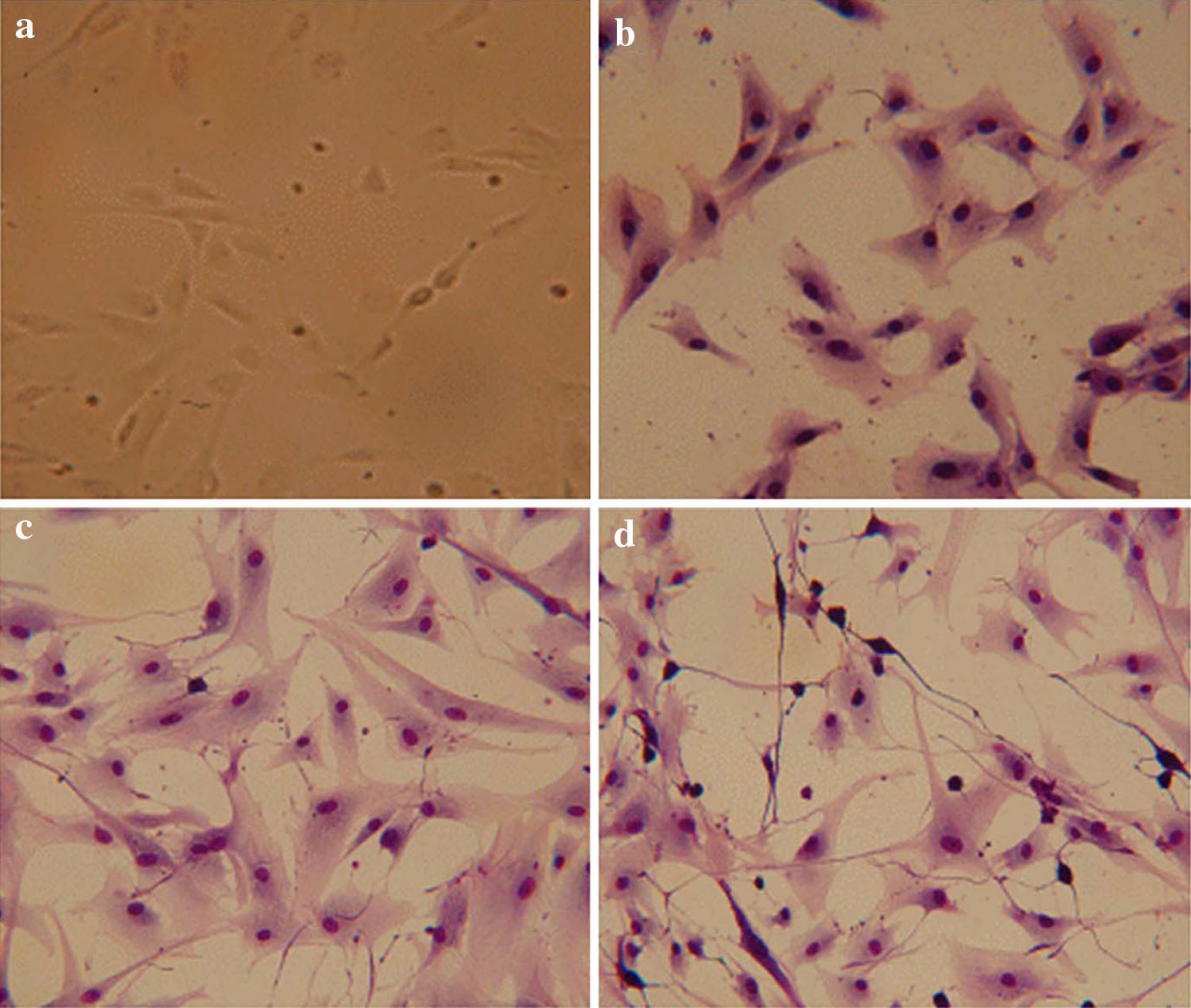
Morphological appearance of neural differentiated canine amniotic fluid mesenchymal stem cells (cAF-MSCs). **a** Before differentiation, cAF-MSCs showed fibroblast-like-shaped cells. **b** Undifferentiated cAF-MSCs stained with Diff-Quick. **c** Spindle shape appeared in cAF-MSCs 2 days after induction of neural differentiation. **d** By the end of neuronal precursor differentiation, cAF-MSCs were stained with Diff-Quick solution and could the progressively acquire neuron-like morphology, a large nucleus, and long processes

In our previous report, we noted that cAF-MSCs can be differentiated into adipocytes, osteocytes, and chondrocytes [31]. Moreover, according to FACS and immunocytometry, cAF-MSCs express mesenchymal markers. In this study, we used confirmed cAF-MSCs to estimate the capacity of differentiation into neuronal precursor cells. cAF-MSCs were induced for 5 days and morphological change was confirmed. cAF-MSCs were prepared in basic culture medium containing 10 % FBS prior to culture (Fig. 1a, b). cAF-MSCs were first cultured for 2 days in predifferentiation medium with N2 supplementation (Fig. 1c) and for 3 days in final-differentiation medium (Fig. 1d). After preinduction, cAF-MSCs began to change morphologically into a spindle shape. Following final neural induction, cAF-MSCs progressively acquired long process extension and neuron-like morphology with a dendritic shape.

### Expression levels of mRNA of neural specific genes on neural cell differentiation

**Fig. 2:**
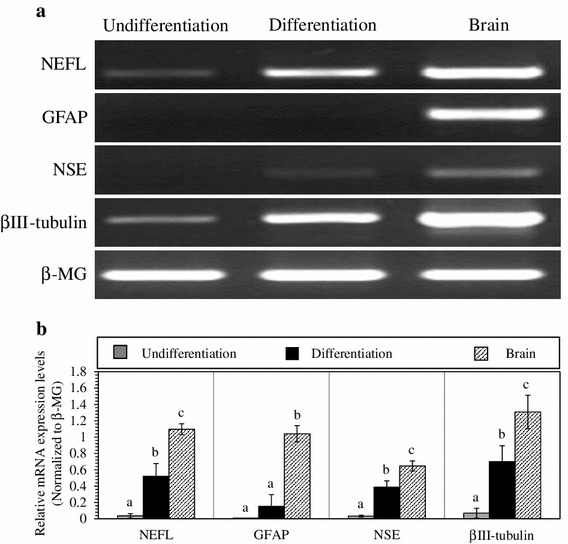
Expression of neuron- and astrocyte-specific markers. **a** *NEFL*,* NSE*,* TUBB3*, and* GFAP* were confirmed by real-time polymerase chain reaction (RT-PCR). βII microglobulin [β-MG (*B2M*)] and brain tissue were used as a housekeeping gene and positive control, respectively. **b** The intensity of messenger RNA (mRNA) expression levels of each gene was quantified and normalized to β-MG

To determine whether cAF-MSCs were induced into neural precursor cells, we tested expression levels of mRNA. RT-PCR results indicated the expression of neural-specific genes, such as* NEFL*,* NSE*,* TUBB3*, and the astrocyte-specific gene,* GFAP*, in neuronal cAF-MSCs before (undifferentiation) and after (differentiation) neural induction. Canine brain tissue was used as a positive control. These genes showed little detectable expression levels of mRNA in the neuronal undifferentiated cAF-MSCs but were significantly increased after induction of neural differentiation (Fig. 2a). Expression levels of* NEFL*,* GFAP*, and* TUBB3* mRNA were much higher in brain tissue. To quantify expression levels of mRNA of each gene, RT-PCR bands were calculated by Image J. Expression level of* NEFL*,* TUBB3*, and* NSE* was significantly increased after neural differentiation, even though* GFAP* was not significantly increased (Fig. 2b). Results showed very similar expression pattern between differentiated cAF-MSCs and mature neural cells.

### Expression levels of neural-specific proteins on neural-cell differentiation

**Fig. 3:**
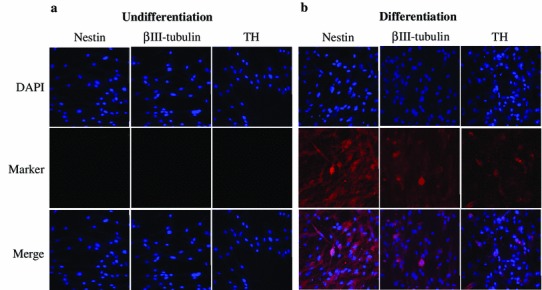
Immunocytochemistry analysis for expression of neuronal-cell-specific markers, such as nestin, βIII-tubulin, and dopamine neuronal-specific marker, tyrosine hydroxylase (TH). Immunocytochemistry was performed in canine amniotic fluid mesenchymal stem cells (cAF-MSCs), which were **a** undifferentiated and **b** differentiated. All neuronal-specific markers were detected in the cytoplasm of differentiated cells, and the nucleus WAS stained by 4′0.6-Diamidino-2-phenylindole (DAPI). Magnification= ×100

Neural-specific protein was analyzed in neuronal undifferentiated and differentiated cAF-MSCs by immunocytochemistry. Protein levels of neuronal-specific markers, such as nestin, βIII-tubulin, and the dopamine neuronal-specific marker, TH, were not expressed in neuronal undifferentiated cAF-MSCs (Fig. 3a). However, protein levels of these markers were dramatically expressed in neuronal differentiated cAF-MSCs, even though TH was not strongly changed in immunocytochemical staining pattern compared with the expression level of undifferentiated cells (Fig. 3b). Interestingly, expression levels of nestin and βIII-tubulin protein were higher than TH in differentiated cAF-MSCs. This result demonstrates that nestin, βIII-tubulin, and TH were only expressed in the cytoplasm and not in nuclei. Furthermore, differentiated neuronal cells can be differentiated to dopamine-related neuronal cells. This indicates that neuronal-specific peptide and protein, as well as dopamine neuronal-specific marker TH, were expressed in differentiated cAF-MSCs.

**Fig. 4:**
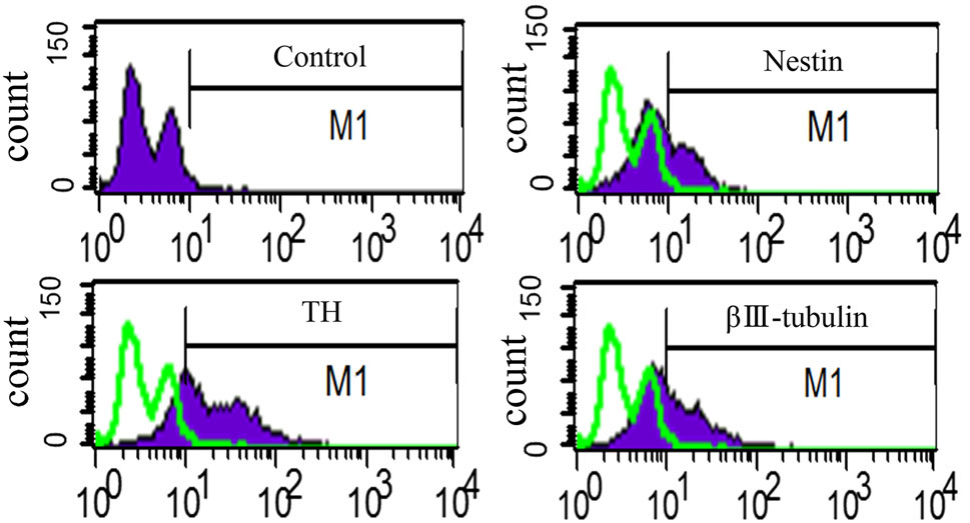
Analysis of expression of neural-specific makers on differentiated neuronal canine amniotic fluid mesenchymal stem cells (cAF-MSCs). Induced neural cAF-MSCs were collected, and III-tubulin, nestin, and tyrosine hydroxylase (TH) expression on their surfaces was measured by flow cytometry. cAF-MSCs:* green-filled histograms* represent staining with isotype-matched antibody

Expression levels of nestin, βIII-tubulin, and TH in differentiated cAF-MSCs were also confirmed after neural differentiation by FACS analysis (Fig. 4). The neuronal-differentiated cAF-MSCs have a greatly increased expression of nestin (32 %), βIII-tubulin (70 %), and TH (43 %) compared with controls (Table 2). After neural differentiation, neural-cell-specific proteins were estimated by FACS.

**Table 2:** Fluorescent-activated cell-sorter (FACS) analysis for expressed proportion of neuronal-cell and dopamine neuronal-cell-specific markers, such as βIII-tubulin, nestin, and tyrosine hydroxylase (TH). The value of each expression level was evaluated after induction

	Control	βIII-tubulin	Nestin	TH
Expression (%)	1.2	69.7	32.22	43.04

## Discussion

In this study, we aimed to investigate whether MSCs originating from cAF-MSC can differentiate into neural precursor cells by identical neural induction reagent. By using N2 supplementation as a neural growth factor, this study was performed in two steps of induction protocol. The first step was preinduction with serum, basic growth factors, and N2 supplementation; the second step was induction with db-cAMP, BHA, and N2 supplementation. This two-step protocol is expected to reduce environmental damage to cells following a change of medium supplementation during induction of cell differentiation.

The intracellular cAMP level plays an important role in the differentiation [12], increasing intracellular cAMP levels, which activates the protein kinase A (PKA) signaling pathway, finally affecting initiate neural differentiation [32]. Previous studies attempted to differentiate neural precursor cells using various reagents, such as db-cAMP [12, 32, 33], neural growth factor [34], brain-derived neural factor [12, 34], 3-isobutyl-1-methylxanthine [12, 32], retinoic acid [12, 32], sonic hedgehog homolog [12, 35], forskolin [36] and 2-mercaptoethanol (β-ME) [37].

The cAF-MSCs have proved their ability to differentiate into neural cells, and in our study, these cells possess the property of dopaminergic neurons. The cAF-MSCs differentiated into neuronal precursor and dopaminergic neurons, with this characteristic being identified though morphology, RT-PCR, immunocytochemistry, and FACS analysis. Dopamine, the catecholamine neurotransmitter, is an important indicator in Parkinson’s diseases. Parkinson’s disease is a progressively neurodegenerative disorder, caused mainly by degenerating dopamine neurons in the nigrostriatal pathway [38]. Therefore, replacement therapy using dopamine neurons could be a potentially successful treatment for patients with Parkinson’s disease [39, 40]. TH is involved in the essential role of dopamine neuron development and is expressed from the hypothalamus, midbrain, brain stem, and olfactory bulb in the central nervous system (CNS). All catecholamine neuron cells, including dopamine neurons, highly express the* TH* gene, which synthesizes catechol as a rate-limiting enzyme [12, 33]. The TH enzyme is also expressed in the amnion [41]. It suggested that amnion-derived stem cells, including AF-MSCs, have the potential to be differentiated into dopaminergic neurons. Undifferentiated AF-MSCs weakly express some neuronal lineage markers, such as neuron, ganglia, catecholamine, dopamine, and neurotrophic factors [23, 42, 43]. These results mean that AF is ready to use as a potential source for the induction of neuronal cells and functional dopaminergic neurons.

The astrocyte, one of three major cell type of the CNS, plays an important role as a supporting neuronal cell. In neuronal and brain injury; however, astrocytes active proliferation rate and changes to the form of reactive gliosis [44]. Pathologically, these astrocytic changes contributed to progression of neuronal degeneration and inflammation, causing neuron degeneration disease [45]. Therefore, inflammatory reaction, such as the occurrence of macrophages and microglia, is related to treatment of neuronal degenerative diseases [46–48]. Analysis using mRNA shows that expression of neuronal-specific genes on neuronal-cell differentiation and GFAP expression level was not significantly increased. Therapeutically, low GFAP expression in undifferentiated and differentiated cAF-MSCs is considered an advantage to this potential source of cell transplantation therapy.

This study demonstrates that cAF-MSCs have the ability to effectively differentiate into neural precursor cells and that cAF-MSCs can be induced into dopamine neuron-like cells with db-cAMP and BHA treatment. Morphologically, after neural induction treatment, cAF-MSCs with a fibroblast-like shape changed to neural-like cells, with long processes, growth cone, dendritic processes, and neuron-network-like structures, as noted in the previous studies [18, 23]. By analyzing protein expression using immunocytochemistry, we corroborated the differentiation potential of cAF-MSCs. These results clearly show that cAF-MSCs could be differentiated into functional neuron precursor cells, in contrast with previous studies [1, 32, 34]. FACS analysis also shows that neural-induced cAF-MSCs indicate the considerable differentiation potential of nestin (32.22 %), βIII-tubulin (69.74 %), and TH (43.04 %).

In conclusion, cAF-MSCs were successfully differentiated into neuronal precursor and dopaminergic neurons at the same time by one neural induction method, with distinction of another method for dopaminergic induction. This study supports the potential capacity of cAF-MSCs for clinical treatment of neuronal precursor-cell transplantation.
